# Retraction: Inhibition of endoplasmic reticulum stress alleviates cigarette smoke-induced airway inflammation and emphysema

**DOI:** 10.18632/oncotarget.28876

**Published:** 2026-04-17

**Authors:** Yong Wang, Zhen-Zhen Wu, Wei Wang

**Affiliations:** ^1^Department of Pneumology, Affiliated Hefei Hospital of Anhui Medical University, Hefei, Anhui, China; ^2^Department of Immunology, Anqing Medical College, Anqing, Anhui, China; ^3^Pharmaceuticals, Roche R&D Center (China), Shanghai, China

**This article has been retracted:** Oncotarget has concluded its investigation of this paper following a retraction request from the corresponding author. The reasons cited for this request were the non-reproducibility of certain *in vitro* data and the mycoplasma contamination of BEAS-2B cells.

The authors did not specify which particular data points were affected and did not respond to requests for clarification from the Scientific Integrity office. Additionally, a request for institutional assistance in this investigation was not fulfilled. Since all authors have agreed to the retraction and signed the retraction agreement letter, the Editorial decision has been made to retract the paper.

Original article: Oncotarget. 2017; 8:77685–77695. 77685-77695. https://doi.org/10.18632/oncotarget.20768

